# Cardiovascular Manifestations of COVID-19: Insights into a Single-Center Experience

**DOI:** 10.1055/s-0041-1731775

**Published:** 2021-08-09

**Authors:** Sara Schukraft, Jean-Luc Magnin, Stéphane Cook

**Affiliations:** 1 Department of Cardiology, Hospital and University Fribourg, Fribourg, Switzerland

**Keywords:** COVID-19, cardiovascular manifestation, troponin

## Abstract

**Background** Since December 2019, an emerging outbreak of novel coronavirus disease 2019 (COVID-19) is caused by the severe acute respiratory syndrome–coronavirus-2 (SARS-CoV-2). The aim of the present report is to describe a population with elevated levels of high-sensitive cardiac troponin T (hs-cTnT) and report on their management during the pandemic of COVID-19.

**Methods** In this retrospective cohort, we collected data from all patients with hs-cTnT levels of >50 ng/mL admitted to Fribourg Hospital between February 15, 2020, and April 15, 2020. The primary diagnosis for troponin elevation was recorded. Echocardiographic, electrocardiographic, and coronary angiographic data were analyzed for signs of myocardial ischemia, infarction, or other cardiomyopathies. In-hospital follow-up was performed for deaths from all causes and for cardiac deaths. Propensity score matching was used in a subgroup analysis to match COVID-19 and non-COVID-19 patients ( *n* = 21 per group).

**Results** Overall, 215 patients with high hs-cTnT levels were enrolled. The median age was 75 [65–83] years and 30% were women. 21 patients (10%) were diagnosed with COVID-19. Of these, acute myocardial injury related to COVID-19 was the most commonly described cardiovascular manifestation during the pandemic peak. Median troponin values were not different between COVID-19 patients and non-COVID-19 patients (94 vs. 137, *p* = 0.14). The number of cardiological examinations was globally low (echocardiography 51% and coronary angiography 52%) in the context of the pandemic. Patients in the COVID-19 group underwent significantly less echocardiographic examinations (19 vs. 55%, *p* ≤ 0.01) and coronary angiographies (5 vs. 58%, *p* ≤ 0.01) than non-COVID-19 patients. Overall mortality in patient with COVID-19 and elevated troponins was very high, as 38% of patients died during hospitalization including 14% for cardiac death. This trend was confirmed in the propensity score–matched analysis.

**Conclusion** Interpretation of troponins during the COVID-19 pandemic was complicated due to the low number of cardiovascular investigations in this context. Follow-up of patients with COVID-19 and cardiovascular events is important to assess their prognosis and to improve their care.

## Introduction

Since December 2019, a large global outbreak of the novel coronavirus disease 2019 (COVID-19) is caused by the severe acute respiratory syndrome-coronavirus-2 (SARS-CoV-2). 1 The pandemic has been spreading in Switzerland since February 2020. 2 COVID-19 most commonly manifests with respiratory illness 3 but cardiovascular involvement has been confirmed. This is due to both the high prevalence of cardiovascular disease in COVID-19 patients and the development of de novo cardiac complications. 4

Several reports have demonstrated the presence of elevated troponin levels in patients with COVID-19, secondary to acute cardiac lesions including acute myocardial infarction (MI), myocardial injury, acute heart failure, and arrhythmias. 5 According to the recommendations of the American College of Cardiology (ACC), clinicians should measure troponin only in cases of specific clinical suspicion, as the existence of few prospective data in COVID-19 patients with troponins makes their interpretation complicated. 6 In response to this review, Chapman and colleagues argued in Circulation that high-sensitivity cardiac troponin is associated with more severe disease and could be an indicator for poorer prognosis in COVID-19 patients. 7

We sought to describe a population with elevated levels of cardiac troponin T (hs-cTnT), report the cardiological investigations performed during patient's hospitalization and analyze their cardiac diagnosis.

## Methods

### Study Population and Data Collection

We retrospectively included in this analysis all patients admitted to Fribourg Hospital with hs-cTnT > 50 ng/L between February 15, 2020, and May 8, 2020. The inclusion period was chosen based on the peak pandemic according to in-hospital quality monitoring data. The hs-cTnT levels and other specific biomarkers were performed according to clinical practice. Indication of further cardiac investigations was performed according to the clinicians. A clinical follow-up was completed for all included patients during the hospital stay. The data collection, analysis, and reporting have been approved by the Institutional Board of the Hospital of Fribourg.

### Clinical Endpoints

The primary diagnosis for troponin elevation was recorded. Laboratory, echocardiographic, electrocardiographic (ECG), and coronary angiographic data were analyzed for signs of myocardial ischemia, infarction, or other cardiomyopathies. Death from any cause and cardiac death were defined using the ARC definition. 8

### Statistical Method

Categorical variables are reported as count and percentages; continuous variables are reported as mean ± standard deviation (SD) or median [interquartile range (IQR): 25–75%]. Normality was assessed by visual inspection of histograms and the computation of Q-Q plots. Continuous variables are analyzed using the Student *t* -test or the Wilcoxon rank-sum test according to their distribution. Categorical variables were compared using Chi-square or Fisher's exact test as appropriate. We performed a propensity-matched analysis to adjust for baseline imbalances on the outcomes. In the matching procedure, we used the caliper-matching approach that randomly selected a COVID-19 patient with a non-COVID-19 patient from the pool of patients within a caliper of ± 0.015 on the propensity score. Statistical analyses were performed with Stata version 14.0 (StataCorp LP, College Station, Texas, United States).

## Results

### Clinical Characteristics

During the inclusion period, 215 patients with elevated troponin T (hs-cTnT) level were enrolled. The median age of the patients was 75 [65–83] years and and 30% were women. The median length of stay was 6 [3–11] days. The rates of prior hypertension, smoking, diabetes and dyslipidemia were 67, 20, 22, and 49% respectively. Table 1 shows the baseline characteristics of all patients.

**Table 1:** Baseline characteristics

	All patients				Propensity score–matched patients		
	All patients ( *n* = 215)	COVID-19 ( *n* = 21)	Non-COVID-19 ( *n* = 194)	*p* -Value	COVID-19 ( *n* = 21)	Non-COVID-19 ( *n* = 21)	*p* -Value
Characteristics							
Male (%)	150 (70)	16 (76)	134 (69)	0.62	16 (76)	12(57)	0.33
Median age (y)	75 [65–83]	77 [73–86]	75 [65–83]	0.04	77 [73–86]	81 [73–86]	0.95
Length of stay (d)	6 [3–11]	8 [3–21]	6 [2–11]	0.20	8 [3–21]	7 [3–10]	0.55
Hypertension	143 (67)	12 (57)	131 (68)	0.34	12 (57)	14 (66)	0.75
Smoking	42 (20)	2 (10)	40 (21)	0.38	2 (10)	3 (14)	1.00
Diabetes	48 (22)	7 (33)	41 (21)	0.27	7 (33)	3 (14)	0.28
Dyslipidemia	105 (49)	6 (29)	99 (51)	0.07	6 (29)	4 (19)	0.72
Family history	21 (10)	0 (0)	21 (11)	0.24	0 (0)	2 (10)	0.49
Previous MI	35 (16)	2 (10)	33 (17)	0.54	2 (10)	5 (24)	0.41
Laboratory values							
Troponins	133 [77–320]	94 [69–194]	137 [77–369]	0.14	94 [69–194]	133 [69–252]	0.36
CRP	27 [35–107]	145 [69–238]	20 [5–70]	< 0.01	145 [69–238]	20 [6–148]	0.04
Creatinine	107 [83–144]	120 [87–209]	105 [82–142]	0.15	120 [87–209]	108 [85–146]	0.37
D-Dimer	1,293 [618–3,255]	4,581 [1,355–21,637]	752 [505–1,830]	< 0.01	4,581 [1,355–21,637]	711 [421–976]	< 0.01
NT-pro-BNP	3,653 [991–110,053]	3,752 [1,726–5,396]	3,650 [991–10,053]	0.98	3,752 [1,726–5,396]	7,196 [732–11,983]	0.84

Abbreviations: COVID-19, novel coronavirus disease 2019; CRP, C-reactive protein; MI, myocardial infarction; NT-pro-BNP, probrain natriuretic peptide.

Note: Continuous variables are expressed as mean ± standard deviation, median [interquartile range] or *n* (%).

### Clinical Investigations

Patients presented a median troponin value of 133 [77–2,487] ng/L. D-dimer level was available in 43 patients with a median value if 1,293 [618–3,255] ng/mL. The biomarker of inflammation as measured by C-reactive protein (CRP) was recorded in 168 patients with a median value of 27 [35–107] mg/L. Probrain natriuretic peptide (NT-pro-BNP) levels were measured in 71 patients with a median of 3,653 [991–110,053] ng/L. Creatinine median levels were 107 [83–144] µmol/L. Of all patients included, 110 (51%) underwent echocardiography with a median ejection fraction (EF) of 55% [47–60%] and 112 (52%) underwent coronary angiography. Only 1% of patients with troponins underwent other cardiac investigations, such as cardiac magnetic resonance imaging or coronary computed tomography, during hospitalization. The overall in-hospital death rate was 13% and cardiac death was 9% ( Table 2 ). This trend was confirmed in a propensity score-matched analysis. ( Tables 1 and 2 )

**Table 2:** In-hospital outcomes

	All patients				Propensity score-matched patients		
Investigations	All patients ( *n* = 215)	COVID-19 ( *n* = 21)	Non-COVID-19 ( *n* = 194)	*p* -Value	COVID-19 ( *n* = 21)	Non COVID-19 ( *n* = 21)	*p* -Value
Echocardiography	110 (51)	4 (19)	106 (55)	<0.01	4 (19)	11 (53)	0.05
LVEF	55 [47–60]	50 [45–55]	55 [47–60]	0.54	50 [45–55]	60 [55–65]	0.13
Coronary angiography	112 (52)	1 (5)	111 (58)	<0.01	1 (5)	12 (57)	< 0.01
Other investigations (cardiac MRI, coronary CT)	3 (1)	0 (0)	3 (2)	1.00	0 (0)	0 (0)	–
In-hospital outcome							
In-hospital death	28 (13)	8 (38)	20 (10)	0.01	8 (38)	0 (0)	< 0.01
In-hospital cardiac death	19 (9)	3 (14)	16 (8)	0.41	3 (14)	0 (0)	0.23

Abbreviations: COVID-19, novel coronavirus disease 2019; CT, computed tomography; LVEF, left ventricular ejection fraction; MRI, magnetic resonance imaging.

### Clinical Diagnosis

#### COVID-19 Subgroup

**Fig. 1:**
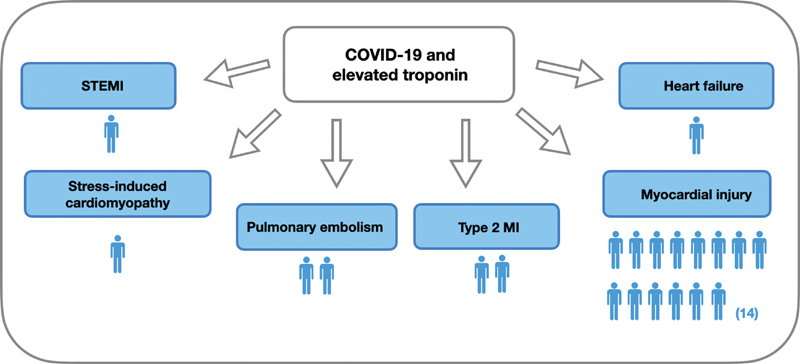
Cardiovascular manifestations of COVID-19 infection during the pandemic peak. CHF, cardiac heart failure; COVID-19, novel coronavirus disease 2019; MI, myocardial infarction; NSTEMI, non-ST-elevation MI; STEMI, ST-elevation MI.

Clinical diagnosis for troponin elevation was variable. COVID-19 was diagnosed in 21 patients (10%). Cardiovascular manifestations in 21 patients with COVID-19 infection were analyzed: 14 patients presented with myocardial injury related to COVID-19, 1 patient presented with ST-segment elevation myocardial infarction (STEMI), 1 patient with stress cardiomyopathy, 2 patients with pulmonary embolism (PE), 2 patients with type-2 MI, and 1 patient with congestive heart failure (CHF; Fig. 1 ).

#### Non-COVID-19 Subgroup

**Fig. 2:**
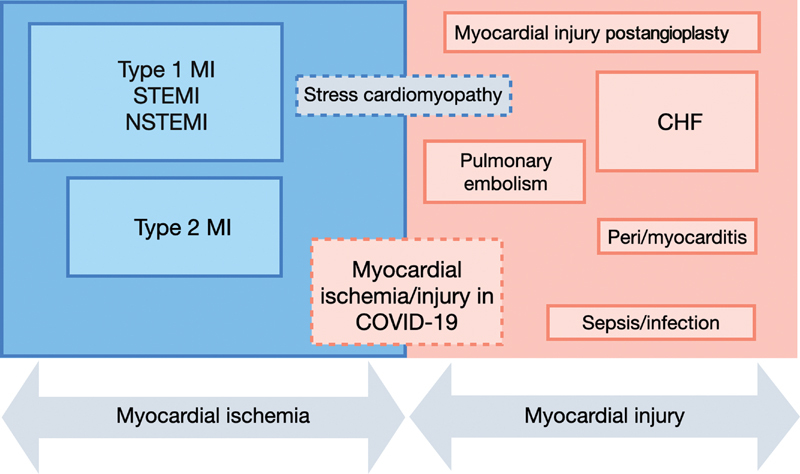
Clinical evidence of acute myocardial ischemia and myocardial injury during the COVID-19 pandemic. COVID, coronavirus disease; MI, myocardial infarction; STEMI, ST-elevation MI.

The World Health Organization (WHO) type-1 MI was the most common diagnosis present in 40% of non-COVID-19 patients including 15% with STEMI and 23% with NSTEMI. The incidence of WHO type-2 MI was 21%. We assessed all clinical diagnoses in Fig. 2 and Table 3 .

**Table 3:** Clinical diagnosis in non-COVID-19 patients with elevated troponin levels during the pandemic peak

Clinical diagnose	*n* = 194
Type-1 MI	77 (40)
NSTEMI	48 (23)
STEMI	29 (15)
Type-2 MI	41 (21)
Myocardial injury in renal failure	27 (14)
Congestive heart failure	17 (9)
Myocardial injury postangioplasty	10 (5)
Myocardial injury in sepsis/infection (non-COVID-19)	7 (4)
Myocardial injury in PE	5 (3)
Takotsubo	3 (2)
Peri-/myocarditis	3 (2)
Other	4 (2)

Abbreviations: COVID-19, novel coronavirus disease 2019; MI, myocardial infarction; NSTEMI, non-ST elevation MI; PE, pulmonary embolism; STEMI, ST elevation MI.

Note: Continuous variables are expressed as mean ± standard deviation, median [interquartile range] or *n* (%).

### COVID-19 versus Non-COVID-19 Patients before Propensity Score Matching

In the COVID-19 subgroup, patients were older than non-COVID-19 patients (77 vs. 75 years, *p* = 0.04; Table 1 ). The median CRP amounts (145 vs. 20 mg/L, *p* < 0.001), as well as the D-dimers (4,581 vs. 752 ng/mL, *p* < 0.01), were higher in the COVID-19 group. Troponins were not significantly different in both groups (94 vs. 137 ng/L, *p* = 0.14). Only four patients underwent echocardiography and one patient underwent coronary angiography in the COVID-19 group, equivalent to a significantly lower median number of examinations than in the non-COVID-19 group (19 vs. 55%, *p* ≤ 0.01 and 5 vs. 58%, *p* ≤ 0.01, respectively). Overall, mortality in the COVID-19 group was very high, as 38% of patients died during hospitalization including 14% for cardiac death.

### COVID-19 versus Non-COVID-19 Patients after Propensity Score Matching

After propensity score–matching analysis, 42 patients were identified, and baseline characteristics of the patients were well balanced between groups ( Table 1 ). Troponin levels remained equivalent between the two groups (94 vs. 133, *p* = 0.36). The results confirm a lower number of echocardiographic examinations (19 vs. 53%, *p* = 0.05) and coronary angiographies (5 vs. 57%, *p* ≤ 0.01) between the matched patients ( Table 2 ).

## Discussion

The main findings of this report are as follows: (1) among patients with elevated troponins during the pandemic peak, 10% were diagnosed with COVID-19; (2) cardiovascular manifestations in patients with COVID-19 infection was varied, ranging from myocardial injury, STEMI, type-2 MI, and stress-induced cardiomyopathy; and (3) in-hospital mortality and cardiac mortality of COVID-19 patients with troponins were high.

### Cardiovascular Manifestations of COVID-19 Infection

This analysis reports a wide and varied panel of cardiac manifestations of COVID-19. Diagnosis of cardiovascular COVID-19 manifestations was performed on a combination of clinical symptoms, specific biomarkers, evidence of new electrocardiographic or echocardiographic abnormalities, or other imaging. Recognition of possible mechanism of cardiovascular manifestations in COVID-19 patients including myocardial injury, plaque rupture or thrombosis (WHO type-1 MI), supply–demand mismatch (WHO type-2 MI), myocarditis, and stress-induced cardiomyopathy is essential for the management and follow-up of these patients.

Acute myocardial injury, defined by an increase in troponins associated or not with electrocardiographic modifications and/or cardiovascular imaging, 9 10 11 was the most commonly described cardiovascular complication in COVID-19 in our center during the pandemic peak which is consistent with recent literature. Potential mechanisms of SARS-CoV-2-mediated myocardial injury are direct myocardial injury of the myocardium or secondary to mechanisms related to an acute systemic inflammatory response (cytokine storm). The overall incidence of acute cardiac injury in COVID-19 patients is variable but estimated between 8 and 12% and is considered an important prognostic marker. 3 12

### Biomarkers

Troponin values were not different between groups. Although troponins are associated with poorer outcomes in patients with COVID-19 according to recent literature, 11 it is interesting to note that the degree of elevation did not differ from a group of patients with non-COVID-related cardiac involvement. Since the pattern of troponin elevation is an essential diagnostic factor, this similarity between troponin values is an additional clinical challenge. Shi et al described a case series of 82 patients with cardiac injury and a median troponin troponin value of 190 ng/L. 10 This higher value of troponin is likely explained by more sever COVID-19 disease in the epicenter of the pandemic with less clinical management experience in the early stages of COVID-19. Finally, recent studies used the 99th percentile as a cut-off point, while our report uses a much higher rule-out threshold (hs-cTnT > 50 ng/L) to avoid uncertain diagnosis for patients in the gray zone.

### Management Implications

Of the 21 COVID-19 patients with troponins, 4 patients (19%) had echocardiography, and 1 patient had a coronary angiography (5%). The reason for the low number of cardiac examinations in COVID-19 patients with troponins is the initially uncertain interpretation of these biomarkers in the COVID-19 context.

Besides, the presence of strict measures to isolate patients and protect health care workers 13 during the peak of the pandemic also contributed to the decrease in the number of tests. Indeed, prolonged and close contact during diagnostic tests (e.g., transthoracic echocardiography, transesophageal echocardiography, and coronary angiography) with these patients was limited. This was necessary to minimize routine diagnostic procedures in a setting where health care resources are already stretched and would also expose health care personnel to an increased risk of exposure to infection.

### Targeted Cardiac Evaluation

To optimize the patient's management, targeted cardiac evaluation was indicated in selected patients with COVID-19 where the evaluation guided the treatment and prognosis. This approach to cardiac assessment may differ from the standard approach, as it is based on weighing the likelihood of the evaluation-guiding decision-making with nosocomial infection control considerations, in a setting with limited availability of medical resources. The level of precautions taken against COVID-19 were differentiated according to the level of risk based on patient presentation and the type of procedures.

The imaging technique was reevaluated on a patient-by-patient basis, both in terms of diagnostic yield and environmental infectious risk. Routine cardiac imaging in patients with suspected or confirmed COVID-19 was reduced to a strict minimum. The management of patients in our center was largely guided by the ESC recommendations for the diagnosis and management of cardiovascular disease during the COVID-19 pandemic. 14 Furthermore, emphasis was placed on a proper triage to favor a correct patient assignment based on the infective status and rapid intervention for patients requiring urgent cardiac intervention with adoption of required protective measures.

### Potential Long-Term Consequences

Considering this, careful follow-up of those recovering from the current COVID-19 would be important to understand the long-term impact of this illness and also to protect these patients from future cardiovascular disease.

## Limitations

There are a number of limitations in the present report. Because of the reduced number of invasive and noninvasive imaging during the COVID-19 pandemic, patients were assessed on clinical evidence, and some mechanisms of cardiac injury may have been misdiagnosed. Moreover, this report is limited to intrahospital outcomes and long-term follow-up has not been performed. The presence of COVID-19 infection and cardiac involvement does not necessarily evoke a direct causal relationship. Further, laboratory values (such as troponin levels, CRP, creatinine, D-dimer, and NT-pro-BNP) were performed according to clinical practice and were not available for all patients which limits generalization of the laboratory results. Finally, outcome analyses in our study should be interpreted with caution due to the small number of patients. Data from larger population and multicenter data are needed to further confirm the implications of cardiac manifestations in COVID-19.

## Conclusion

This report displays a large panel of possible cardiovascular manifestations of COVID-19. The presumed pathophysiological processes provide a better understanding of these cases but many may have multifactorial etiologies. However, clinical diagnosis was challenged by a reduction of cardiological investigations during the pandemic.

## References

[1] Chen L Liu W Zhang Q RNA based mNGS approach identifies a novel human coronavirus from two individual pneumonia cases in 2019 Wuhan outbreak Emerg Microbes Infect 2020 9 01 313 319 32020836 10.1080/22221751.2020.1725399 PMC7033720

[2] Federal Office of Public Health New Coronavirus 2019-nCoV: first confirmed case in SwitzerlandAccessed June 10, 2020 at:https://www.bag.admin.ch/bag/en/home/das-bag/aktuell/medienmitteilungen.msg-id-78233.html

[3] Huang C Wang Y Li X Clinical features of patients infected with 2019 novel coronavirus in Wuhan, China Lancet 2020 395(10223):497 506 31986264 10.1016/S0140-6736(20)30183-5 PMC7159299

[4] Clerkin K J Fried J A Raikhelkar J COVID-19 and cardiovascular disease Circulation 2020 141 20 1648 1655 32200663 10.1161/CIRCULATIONAHA.120.046941

[5] Long B Brady W J Koyfman A Gottlieb M Cardiovascular complications in COVID-19 Am J Emerg Med 2020 38 07 1504 1507 32317203 10.1016/j.ajem.2020.04.048 PMC7165109

[6] American College of Cardiology Januzzi J L Jr. Troponin and BNP use in COVID-19Accessed June 10, 2020 at:https://www.acc.org/latest-in-cardiology/articles/2020/03/18/15/25/troponin-and-bnp-use-in-covid19

[7] Chapman A R Bularga A Mills N L High-sensitivity cardiac troponin can be an ally in the fight against COVID-19 Circulation 2020 141 22 1733 1735 32251612 10.1161/CIRCULATIONAHA.120.047008

[8] Academic Research Consortium Cutlip D E Windecker S Mehran R Clinical end points in coronary stent trials: a case for standardized definitions Circulation 2007 115 17 2344 2351 17470709 10.1161/CIRCULATIONAHA.106.685313

[9] Guo T Fan Y Chen M Cardiovascular implications of fatal outcomes of patients with coronavirus disease 2019 (COVID-19) JAMA Cardiol 2020 5 07 811 818 32219356 10.1001/jamacardio.2020.1017 PMC7101506

[10] Shi S Qin M Shen B Association of cardiac injury with mortality in hospitalized patients with COVID-19 in Wuhan, China JAMA Cardiol 2020 5 07 802 810 10.1001/jamacardio.2020.095010.1001/jamacardio.2020.0950 32211816 PMC7097841

[11] Zhang Y Chen H Cao B Clinical course and risk factors for mortality of adult inpatients with COVID-19 in Wuhan, China: a retrospective cohort study Lancet 2020 135 2033 2040 10.1016/S0140-6736(20)30566-3 PMC7270627 32171076

[12] Wang D Hu B Hu C Clinical characteristics of 138 hospitalized patients with 2019 novel coronavirus-infected pneumonia in Wuhan, China JAMA 2020 323 11 1061 1069 32031570 10.1001/jama.2020.1585 PMC7042881

[13] World Health Organization Rational use of personal protective equipment for coronavirus disease (COVID-19): interim guidance, 27 February 2020Accessed February 27, 2020 at:https://apps.who.int/iris/handle/10665/331215

[14] ESC Guidance for the Diagnosis and Management of CV Disease during the COVID-19 PandemicAccessed June 10, 2020 at:https://www.escardio.org/Education/COVID-19-and-Cardiology/ESC-COVID-19-Guidance

